# Supplementary material for: Sustained increase in suspended sediments near global river deltas over the past two decades
Source: Nat Commun. 2024 Apr 18;15:3319. doi: 10.1038/s41467-024-47598-6 (PMC11026514; doi:10.1038/s41467-024-47598-6)
Supplement: Supplementary file 1 — Supplementary Information [file 41467_2024_47598_MOESM1_ESM.pdf]

Supplementary information

Sustained increase in suspended sediments near global river deltas over the past two decades

Supplementary Figures 1-14

Supplementary Tables 1-3

Supplementary Notes 1

Supplementary Figures

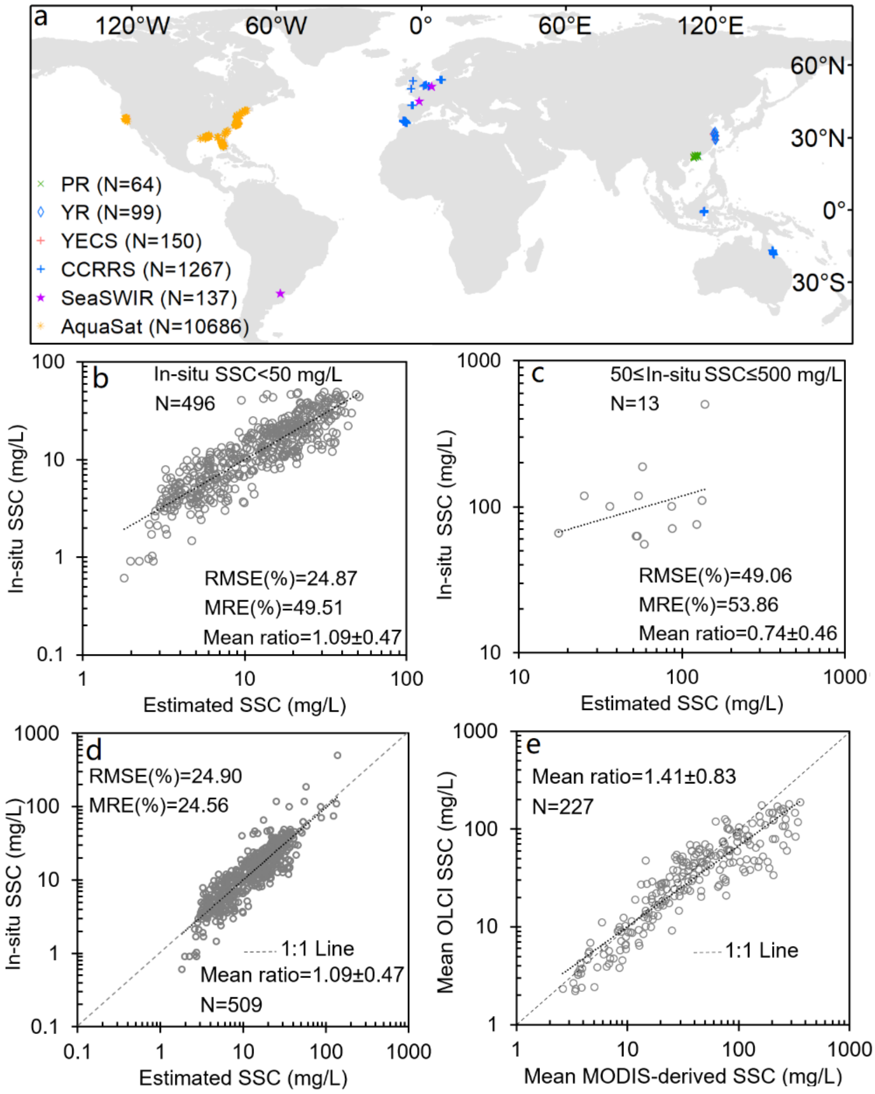

Supplementary Figure 1 | Sampling locations of in-situ suspended sediment concentration (SSC) and validation of SSC inversion models used in this study. (a). Locations of the in-situ SSC datasets used in this study. The number of samples utilized in each dataset is indicated in brackets; **(b-d).** Accuracy assessments of applying the SSC inversion model used in this study to clear (< 50 mg/L) (b) and turbid water (50-500 mg/L) (c), as well as the total accuracy of this model (d). The mean ratio between in-situ SSC and MODIS-derived SSC is annotated; **(e).** A comparison between the multi-year (2016-2020) average MODIS-derived SSC (500 m resolution) in this study and the OLCI-derived SSC (4 km resolution).

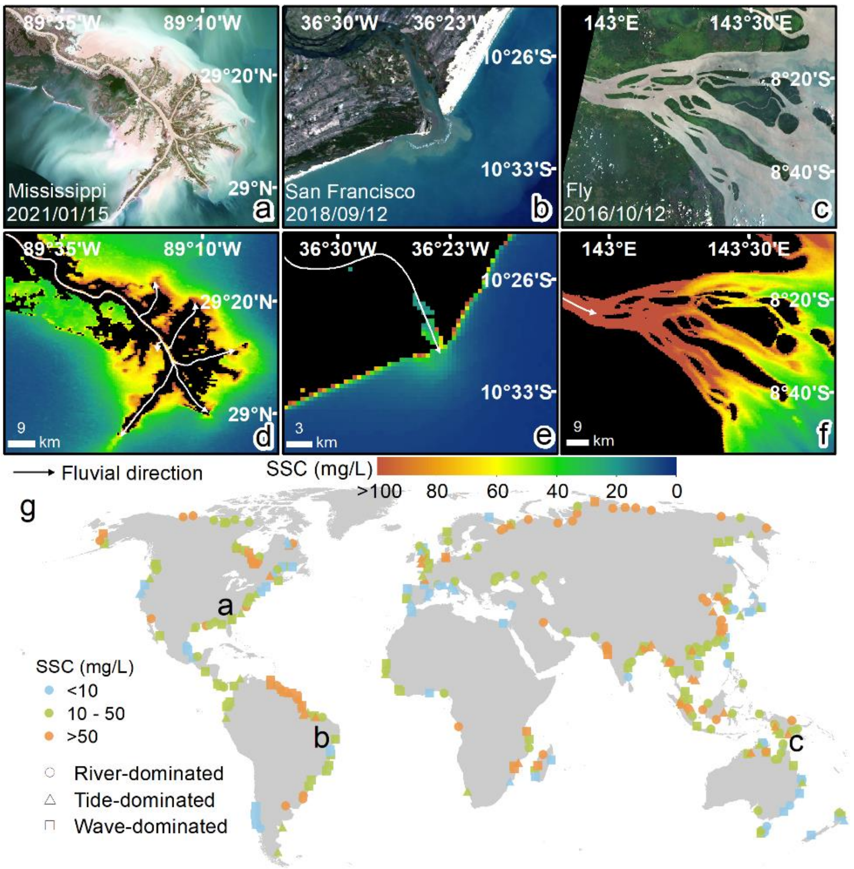

Supplementary Figure 2 | Long-term mean suspended sediment concentration (SSC) for 349 deltas with different delta morphologies. **(a-f)**. Examples of SSC for three delta morphologies, including river-dominated (Mississippi, USA), wave-dominated (San Francisco, Brazil), and tide-dominated (Fly, New Guinea). True color images collected from Landsat 8 (a-c) and the corresponding long-term mean MODIS-derived SSC(d-f) were displayed; **(g)**. Locations of 349 deltas with different morphologies were included in our study.

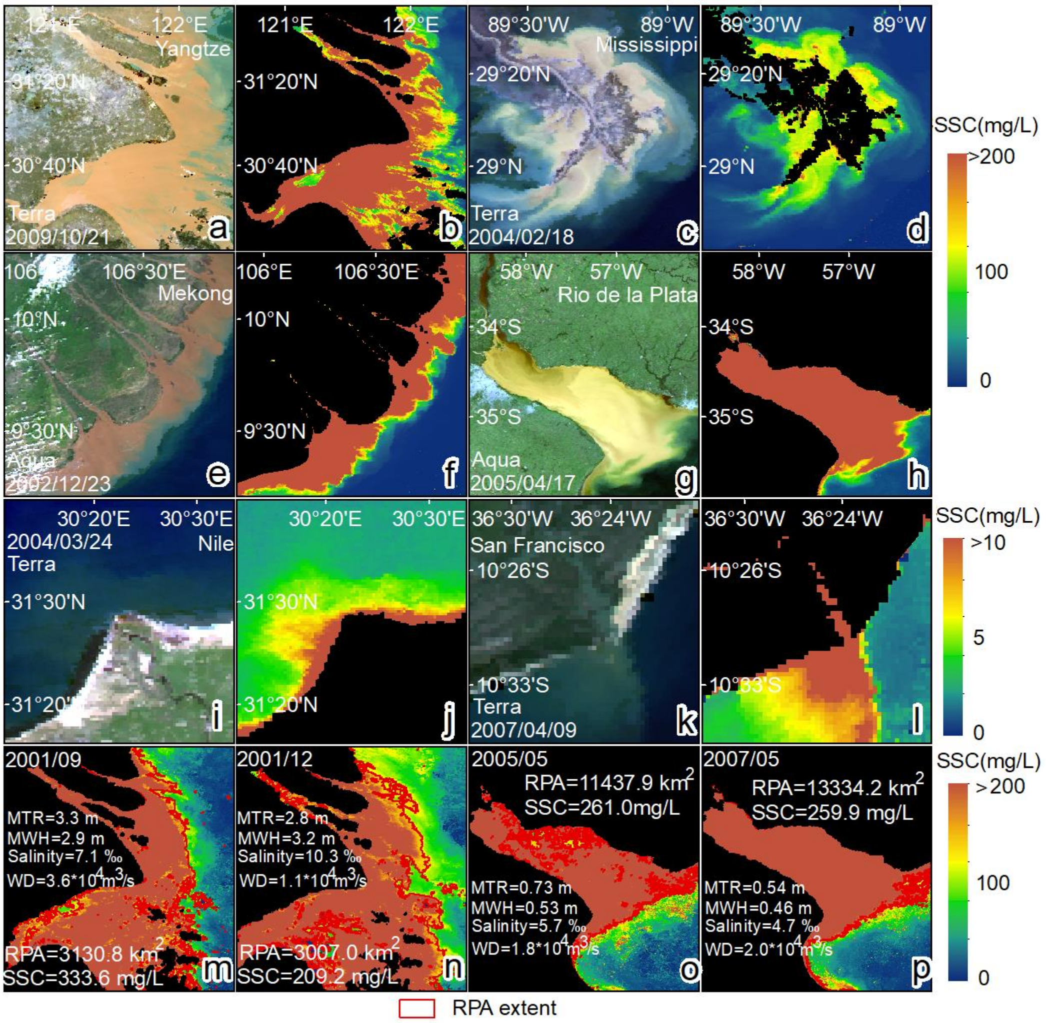

Supplementary Figure 3 | Spatial distributions of suspended sediment concentration (SSC) generated in this study and the spatial variations of river sediment plume area (RPA, unit: km^2) in various deltas. (a-l). Spatial distributions of SSC in the following rivers: Yangtze, China (a-b), Mississippi, USA (c-d), Mekong, Vietnam (e-f), Rio de la Plata, Argentina (g-h), Nile, Egypt (i-j), and San Francisco, Brazil (k-l). True color images and SSC were derived from MODIS 8-day surface reflectance products. The MODIS sensors (Terra or Aqua), image dates, and delta names are annotated; **(m-p).** Comparisons of variations of monthly RPA and mean SSC in Yangtze and Rio de la Plata at different periods with different hydrodynamics. The RPA (red polygon) is defined as the extent where the monthly mean SSC exceeds a specific SSC threshold (Methods, Extended Data Fig. 14) determined for each river mouth. The time, RPA, mean SSC, along with monthly maximum tidal range (MRT, unit: in m), maximum wave height (MWH, unit: in m), mean salinity (unit: in %), and mean water discharge (WD, unit: m^3/s) was tagged.

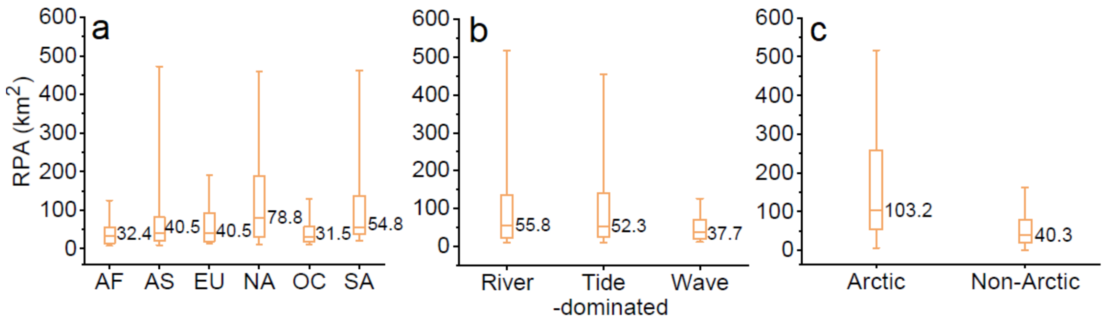

Supplementary Figure 4 | Box plots of long-term mean RPA (river sediment plume area, unit: km^2) in different continents (AF: Africa; AS: Asia; EU: Europe; NA: North America; OC: Oceania; SA: South America), different delta morphologies, and Arctic (latitude >50°N) and non-Arctic deltas. The RPA indicates the extent where the monthly mean suspended sediment concentration (SSC) over a specific SSC threshold (see Methods). The box plots in (a-c) show the distributions (10, 25, 50, 75, and 90%) of RPA.

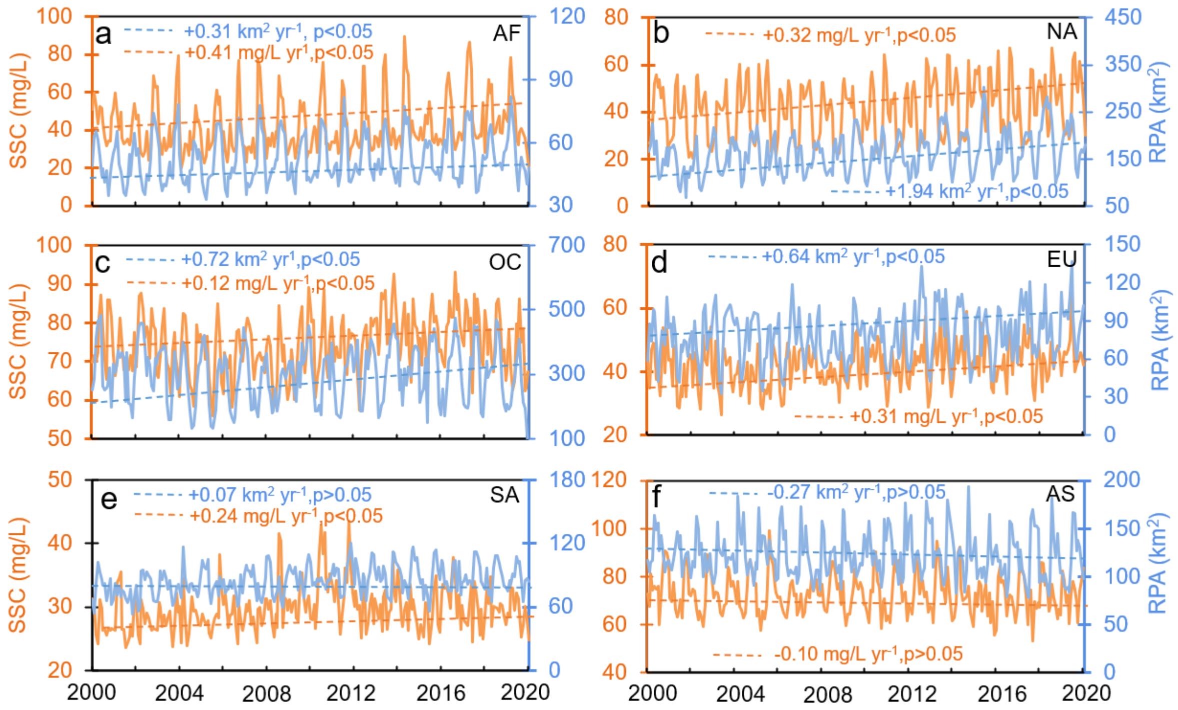

Supplementary Figure 5 | Long-term trends in the delta coastal suspended sediment concentration (SSC) and river sediment plume area (RPA) across six continents. (a-f). Variations in monthly mean SSC and RPA , along with their long-term change trends. The orange and blue dash lines represent the long-term trends of SSC and RPA, respectively. These trends were derived based on long-term monthly anomaly SSC and RPA, which were estimated as the difference between the monthly mean SSC and RPA and their long-term average for that month (see Methods). The Sen's slope and *p*-value obtained from the Mann-Kendall test are annotated. AF: Africa; AS: Asia; EU: Europe; NA: North America; OC: Oceania; SA: South America.

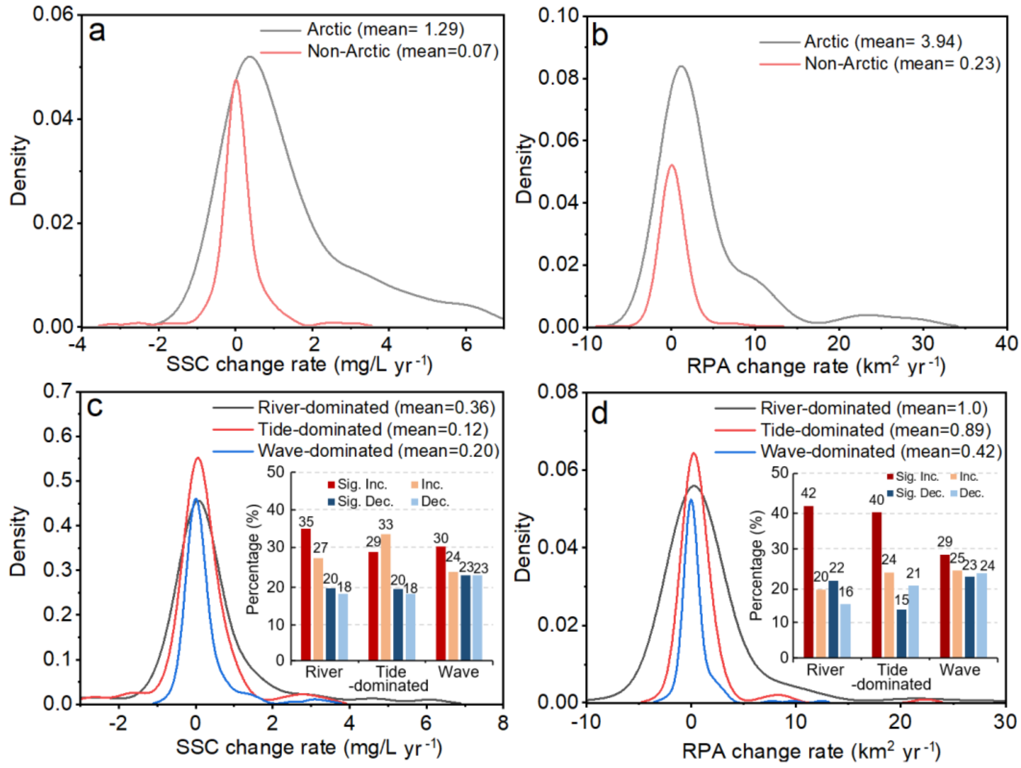

Supplementary Figure 6 | Comparison of the change rates of suspended sediment concentration (SSC) and river sediment plume area (RPA) between the Arctic (latitude >50° N) and non-Arctic river deltas, as well as different delta morphologies. (a-b). Density histograms of the change rates (Sen's slope from Mann-Kendall test) of SSC (a) and RPA (b) in Arctic and non-Arctic river deltas; **(c-d)**. Density histograms of the change rates of SSC (a) and RPA (b) among different delta morphologies. The bar chart in the panel illustrates the percentage of deltas presenting different change trends (significant increase or decrease, increase or decrease). The mean change rates for SSC and RPA are indicated in parentheses following the legend. All plots were generated using Origin 2021.

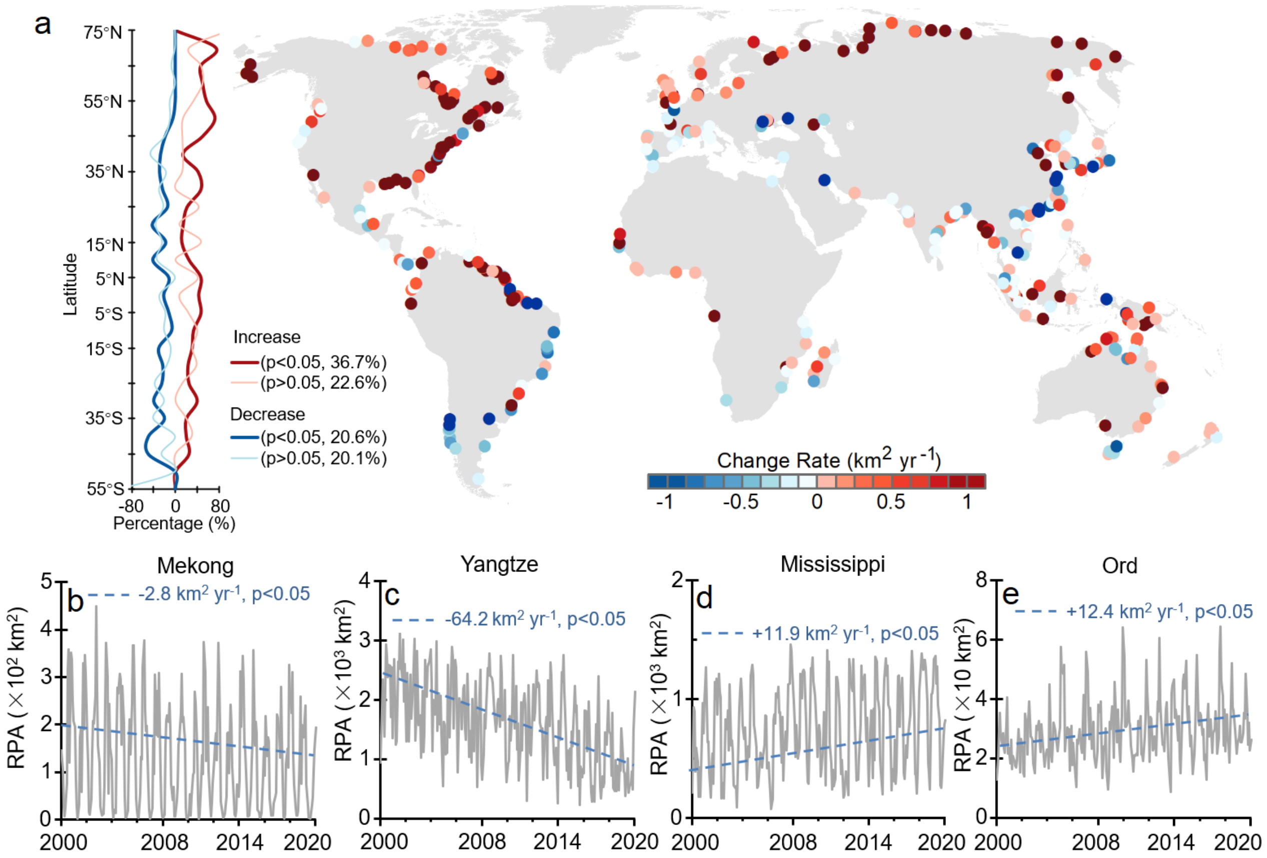

Supplementary Figure 7 | Trends of global coastal river sediment plume area (RPA) between 2000 and 2020. (a). Spatial patterns of the RPA trends (Mann-Kendall test, method) in 349 deltas. The latitudinal profiles exhibit the percentages of deltas with significant (p<0.05) and insignificant RPA change trends (increase or decrease); **(b-c).** The monthly mean variations of RPAs and their long-term trends for Mekong (b), Yangtze (c), Mississippi (d), and Ord (e). The blue dash line represents the long-term trend of RPA. This trend was derived based on long-term monthly anomaly RPA, calculated as the difference between the monthly mean RPA and its long-term average for that month (see Methods). The Sen's slope and p-value obtained from the Mann-Kendall test are annotated.

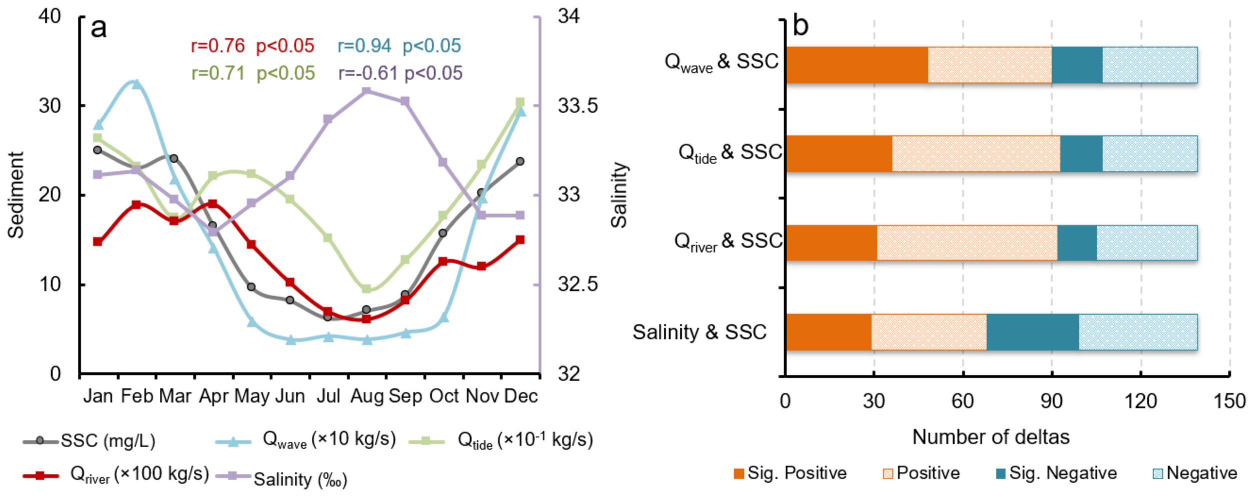

Supplementary Figure 8 | Relationships between long-term monthly mean suspended sediment concentration (SSC), Q_{river}, Q_{tide}, Q_{wave}, and salinity. (a). Correlation relationships between seasonal variations of SSC and Q_{river}, Q_{tide}, Q_{wave}, and salinity for the Ramu delta, Papua New Guinea. The Pearson correlation coefficients (*r*) and *P* values (*p*) were annotated; **(b).** Statistics for significant and insignificant correlation relationships (positive or negative) between long-term monthly mean SSC and Q_{river}, Q_{tide}, Q_{wave}, and salinity for 139 deltas. The correlation analysis was conducted using R 3.3.0.

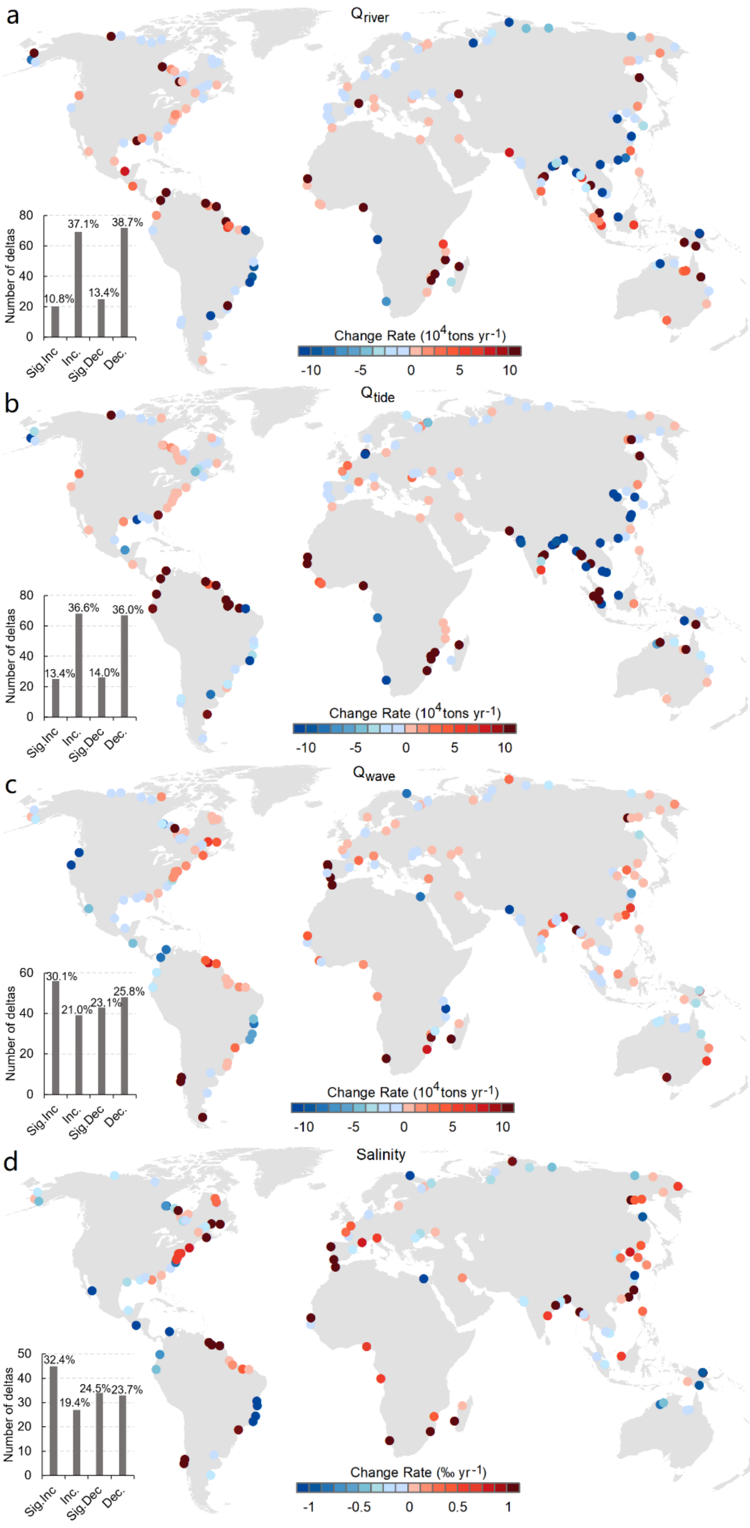

Supplementary Figure 9 | Long-term changes of Q_{river}, Q_{tide}, Q_{wave}, and salinity of global deltas. **(a-c).** Distribution of long-term annual changes of Q_{river}, Q_{tide}, and Q_{wave} in 186 river deltas. Trends were analyzed from 2000 to 2020; **(d).** Long-term variations of salinity for 139 deltas from 2000 to 2020. The change rate is represented by Sen's slope from the Mann-Kendall test. The left chart of each panel displays the number of deltas with different changing trends. Sig. Inc.: significant (p<0.05) increase; Sig. Dec.: significant (p<0.05) decrease; Inc.: Increase; Dec.: Decrease.

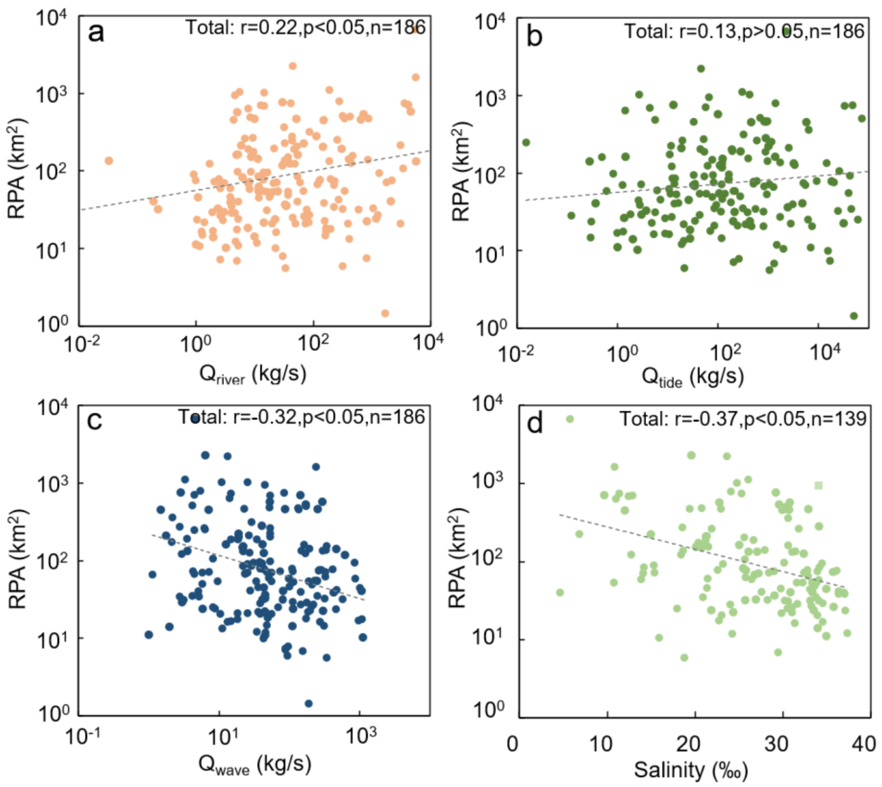

Supplementary Figure 10 | Relationships between long-term mean river sediment plume area (RPA), Q_{river}, Q_{tide}, Q_{wave}, and salinity. (a-d). The relationships between RPA and (a) Q_{river}, (b) Q_{tide}, (c) Q_{wave}, and (d) salinity. The number of deltas (n), correlation coefficients (*r*) and *P* values (*p*) were annotated. The correlation coefficients are based on the logarithm-transformed RPA and different factors. The relationships between RPA and $Q_{river,}$ Q_{tide}, and Q_{wave} were conducted for 189 deltas, while due to the data availability, relationships between RAP and salinity were only conducted for 139 deltas. The correlation analysis was conducted using R 3.3.0.

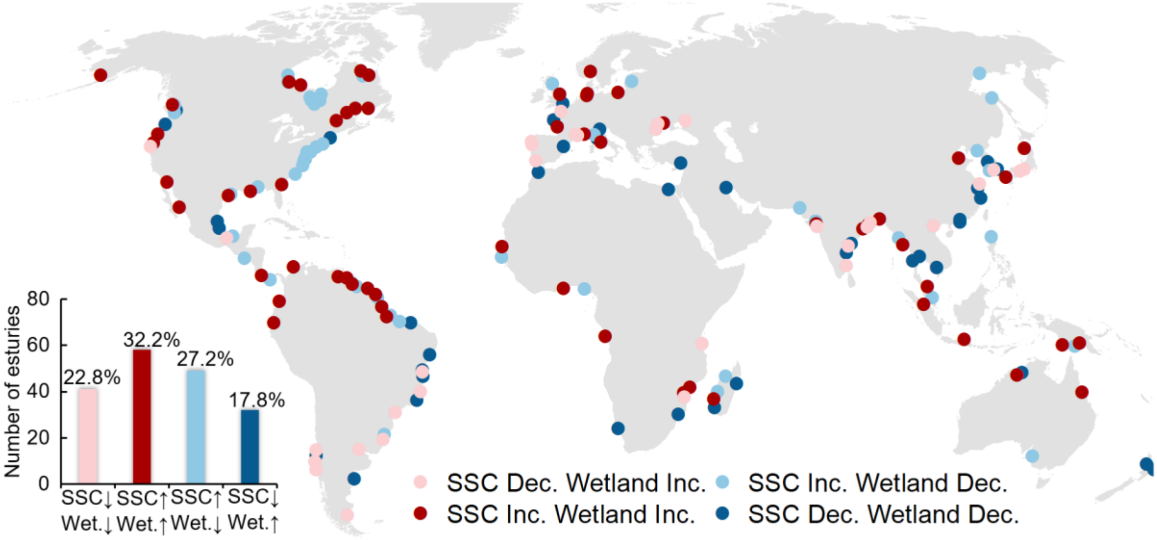

Supplementary Figure 11 | Change trends of wetland and delta coastal suspended sediment concentration (SSC) between 2000 and 2020 for 180 deltas. Spatial patterns of the trends (increase or decrease) in wetland areas and delta coastal SSC. Due to the availability of wetland data, only 180 deltas were examined. The left bar chart shows the counts of deltas with different combinations of changing trends between SSC and wetland areas. The upward arrow (' ↑ ') represents an increasing trend, while the downward arrow(' ↓ ') indicates a decreasing trend.

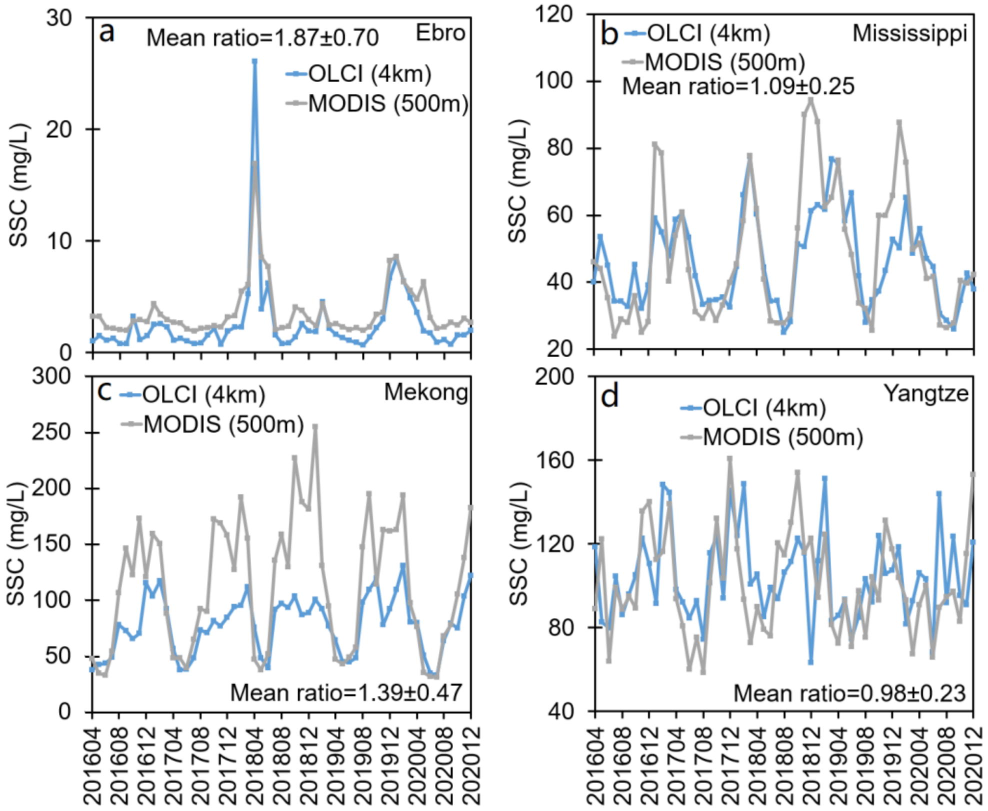

Supplementary Figure 12 | Comparisons of monthly mean time series (from April 2016 to December 2020) between MODIS-derived suspended sediment concentration (SSC) in this study and the corresponding OLCI-derived SSC among different river deltas (ranging from clear to turbid). The mean ratio in (a-d) represents the ratio of MODIS SSC and OLCI SSC.

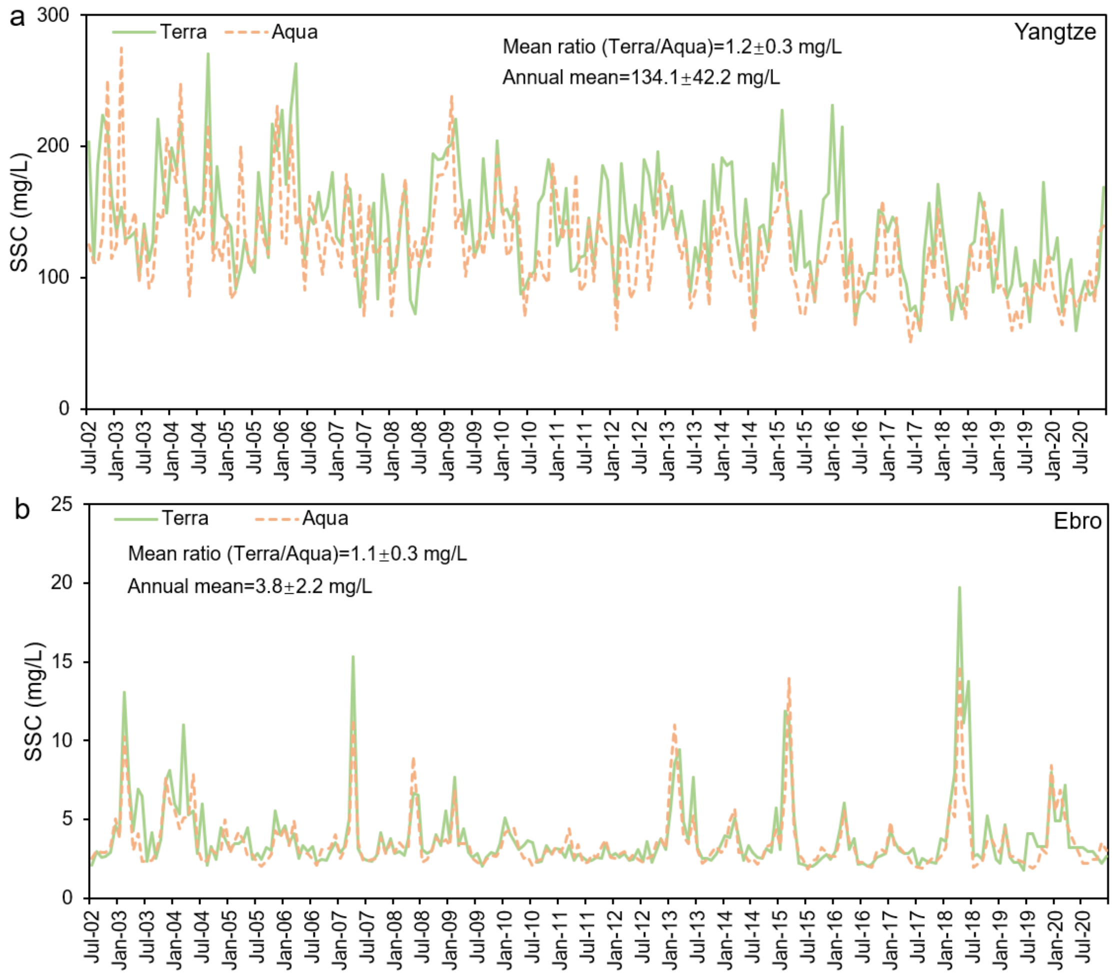

Supplementary Figure 13 | Comparison of monthly mean suspended sediment concentration (SSC) in the Yangtze and Ebro based on the Yu_adapted algorithm and MODIS Terra and Aqua surface reflectance data. The mean and standard deviation of the monthly mean ratios between Terra and Aqua, as well as the multi-year average SSC for both estuaries, are provided.

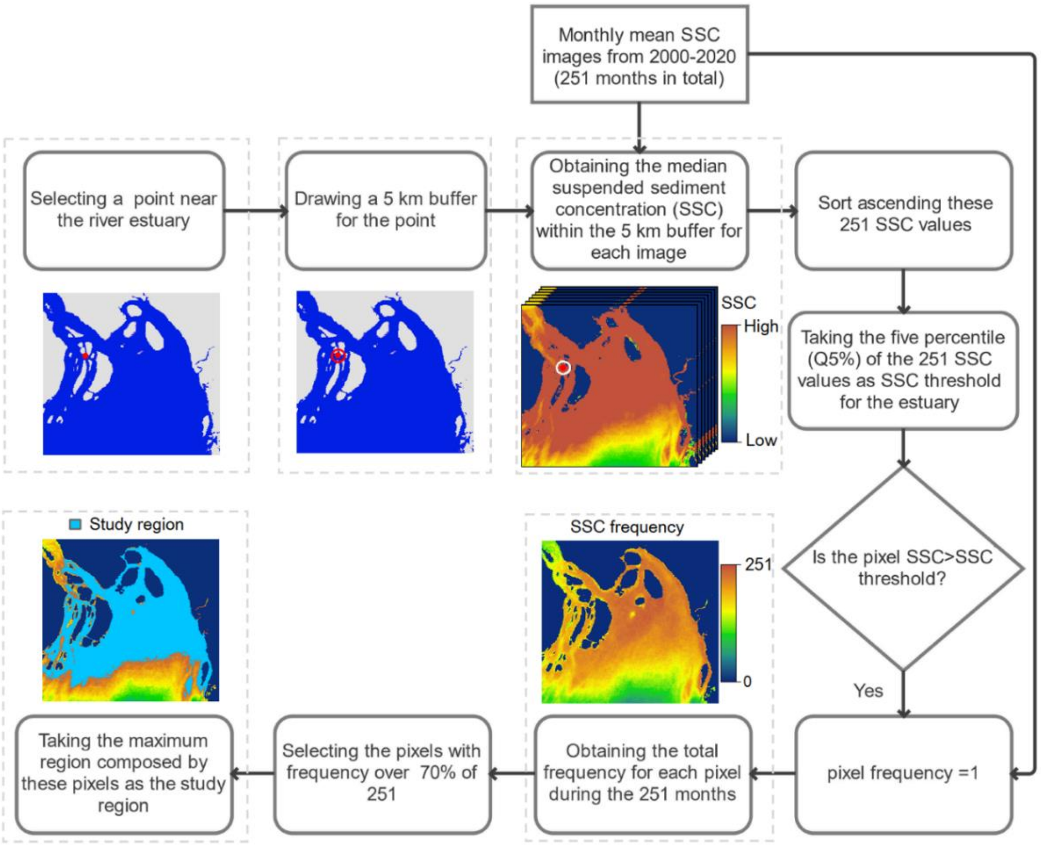

Supplementary Figure 14 | Flow chart for study region determination near deltaic coast. Working steps in the determination of coastal study region near delta based on MODIS derived suspended sediment concentration (SSC).

Supplementary Tables

Supplementary Table 1 | Accuracy of the Han_adapted (adapted from Han et al., (2016)), Feng_adapted (adapted from Feng et al., (2014)) and Yu_adapted (adapted from Yu et al., (2019)) algorithm used in this study at various suspended sediment concentration (SSC) levels. The number (n) in parentheses represents the sample size.

	Han_adapted		Feng_adapted		Yu_adapted	
	RMSE(%)	MRE(%)	RMSE(%)	MRE(%)	RMSE(%)	MRE(%)
SSC<50 mg/L (n=496)	63.23	78.53	28.51	53.68	24.87	49.51
500>=SSC>=50 mg/L (n=13)	68.94	60.32	48.64	64.28	49.06	53.86
Total	56.04	51.93	28.64	29.08	24.90	24.56

Supplementary Table 2 | Comparison of the ranges of SSC derived by MODIS in this study with the suspended sediment concentration (SSC) collected from other publications for 31 delta coasts. The SSC ranges in this study represent the minimum and maximum monthly mean SSC from 2000-2020, while the SSC ranges in others are the records collected from existing publications.

Name	This study			Others			
	Dates	Sensors	SSC (mg/L)	Dates	Sensors/In situ	SSC (mg/L)	References
Ebro	2000.02-2020.12	MODIS	1.8-16.9	1997.06-10	In situ	0.98-2.8	Lahet et al. (2000, 2001)
Adour	2000.02-2020.12	MODIS	2.1-33.0	2007.7.14	In situ	0.3-145.6	Petus et al. (2010)
Atchafalaya	2000.02-2020.12	MODIS	27.1-305.1	1980-2015	In situ	133.0-249.0	Mize et al. (2018)
Apalachicola	2000.02-2020.12	MODIS	11.8-201.8	2008.10.11	MODIS	2.5-67.8	Chen et al. (2011)
Arno	2000.02-2020.12	MODIS	1.9-18.8	1991.1	Landsat	0.0-36.0	Nicolini et al. (1999)
Chikugo	2000.02-2020.12	MODIS	15.7-55.2	2010.09	In situ	37.2-1249.6	Azhikodan et al. (2016)
Danshui	2000.02-2020.12	MODIS	2.2-20.2	1997.11	In situ	39.0	Xu et al. (2012)
Rhone	2000.02-2020.12	MODIS	2.4-50.8	1994.11;2010.03	SPOT; MERIS	0.5-40.0	Ouillon et al. (1997)<br>Lorthiois et al. (2012)
Orinoco	2000.02-2020.12	MODIS	73.3-252.8	2001-2016	MODIS	11.0-290.0	Gallay et al. (2019)
Oujiang	2000.02-2020.12	MODIS	46.2-391.2	2009.10.14	In situ	10.0-320.0	Chen et al. (2013)
San Francisco	2000.02-2020.12	MODIS	4.4-51.0	2000.07&12;<br>2001.02&09	Landsat	6.5-12.6	Olivera et al. (2012)

Indus	2000.02-2020.12	MODIS	3.7-365.5	2001.11;<br>2005.03;<br>2016.03	Landsat	0.0-300.0	Shahzad et al. (2018)
Rhine	2000.02-2020.12	MODIS	9.3-64.0	2014.09-10	In situ	10.0-50.0	Flores et al. (2017)
Vistula	2000.02-2020.12	MODIS	7.4-67.9	2010.03 &11	In situ	4.0-16.0	Szymczak et al. (2013)
Pearl River	2000.02-2020.12	MODIS	13.7-75.2	2003-2015	MODIS	0.0-100.0	Zhang et al. (2018)
Fraser	2000.02-2020.12	MODIS	6.2-106.9	1988.05&06	In situ	53.0-190.0	Kostaschuk et al. (1993)
Magdalena	2000.02-2020.12	MODIS	6.2-47.4	2003-2017	MODIS	99.9-257.1	Torregroza-Espinosa et al. (2020)
Mississippi	2000.02-2020.12	MODIS	17.1-111.7	1989.03 & 04 & 10;<br>2011.04-07	AVHRR; MODIS	10.0-3500.0	Walkere et al. (1996);<br>Falcini et al. (2012)
Chao Phraya	2000.02-2020.12	MODIS	13.6-91.2	1983	In situ	10.0-500.0	Dalai et al. (2005);<br>Cheevaporn et al. (2003)
Hooghly	2000.02-2020.12	MODIS	12.5-275.1	2003-2018	MODIS	50.0-250.0	Jayaram et al. (2020)
Huanghe	2000.02-2020.12	MODIS	6.28-291.5	2000-2010	Landsat; MODIS	1.0-500.0	Zhang et al. (2014)
Thames	2000.02-2020.12	MODIS	11.8-88.3	2008-2009	MODIS	10.0-100.0	Vanhellemont et al. (2014)
Rio de la Plata	2000.02-2020.12	MODIS	43.0-474.0	2002-2012	MODIS	10.0-100.0	Moreira et al. (2013)
Fly	2000.02-2020.12	MODIS	33.5-156.2	1992	In situ	500.0-<br>3000.0	Wolanski et al. (1996)
Weser	2000.02-2020.12	MODIS	29.8-190.2	2009.08;2010.03	In situ	28.0-3060.0	Papenmeier et al. (2012)
Ganges	2000.02-2020.12	MODIS	19.2-166.1	1989.01;1991.07 &09	Landsat	200.0-<br>1710.0	Islam et al. (2002)

Mekong	2000.02-2020.12	MODIS	31.6-450.5	2014-2015	Lamdsat; MODIS; RapidEye	50.0-300.0	Wackerman et al. (2017)
Amazon	2000.02-2020.12	MODIS	3.6-416.4	2000-2013	MODIS	150.0-600.0	Gensac et al. (2016)
Mahi	2000.02-2020.12	MODIS	144.0-497.5	——	In situ	2.7-1200.0	Mitra et al. (2021)
Yangtze	2000.02-2020.12	MODIS	36.1-333.6	2004-2008	MERIS	300.0-500.0	Shen et al. (2010)
Yangon	2000.02-2020.12	MODIS	57.5-462.2	2002.05	Oceansat-1	15.0-500.0	Ramaswamy et al. (2004)

Supplementary Table 3 | Correlation analyses and multiple general linear model (GLM) analyses between suspended sediment concentration (SSC) and different driving factors in 139 river deltas. The correlation coefficients between SSC and four drivers (Q_{river}, Q_{tide}, Q_{wave}, and Salinity) and the contributions of these four drivers to SSC changes were listed.

River name	Lat.	Lon.	Continents	Type	Correlation coefficients				Mean Sq					Contributions (%)				
					Qriver	Qtide	Qwave	Salinity	Qriver	Qtide	Qwave	Salinity	Residuals	Qriver	Qtide	Qwave	Salinity	Residuals
Baitarani	20.79	86.99	Asia	river	0.00	-0.13	0.07	0.19	457.69	42.34	38.55	9.44	94.72	71.21	6.59	6.00	1.47	14.74
Batang Hari	-0.97	104.22	Asia	river	0.27	-0.25	-0.63*	0.20	112.76	130.49	64.07	380.64	58.32	15.11	17.49	8.59	51.00	7.81
Betsiboka	-15.84	46.28	Africa	river	0.32	0.30	0.02	0.03	2015.60	292.90	0.46	50.75	1075.19	58.68	8.53	0.01	1.48	31.30
Daugava	57.12	24.02	Europe	river	-0.05	-0.08	-0.06	-0.25	2.79	63.40	72.63	0.03	63.05	1.38	31.40	35.97	0.01	31.23
Dnipro	46.52	32.23	Europe	river	0.23	0.25	-0.15	-0.13	4.29	0.53	1.38	0.01	4.82	38.95	4.81	12.50	0.05	43.69
Don	47.15	39.19	Europe	river	0.51*	0.31	0.52*	-0.15	89.48	1.20	8.15	58.77	12.66	52.55*	0.70	4.79	34.52	7.43
Dong Nai	10.37	106.82	Asia	tide	0.07	0.09	-0.01	0.61*	0.03	4.17	308.79	0.12	31.71	0.01	1.21	89.55	0.04	9.19
Ebro	40.71	0.76	Europe	river	0.39*	0.32	-0.26	0.09	2.70	0.19	0.09	0.50	0.92	61.40	4.26	2.05	11.38	20.91
Ehncen	70.83	83.44	Asia	river	-0.08	-0.09	0.19	-0.30	17.37	2.96	220.32	154.57	129.26	3.31	0.56	42.01	29.47	24.65
Elbe	53.9	8.84	Europe	tide	0.02	0.36	-0.04	-0.15	1.70	443.99	24.53	267.78	166.41	0.19	49.09	2.71	29.61	18.40
Ganges	22.8	90.72	Asia	tide	0.27	0.30	-0.04	0.11	25.49	10.62	0.36	0.07	19.20	45.72	19.05	0.65	0.13	34.44
Godavari	16.75	81.9	Asia	river	-0.10	-0.12	0.17	0.17	2.73	13.77	14.40	8.83	16.26	4.87	24.59	25.72	15.77	29.05
Haihe	38.97	117.78	Asia	river	0.79*	0.72*	-0.24	0.54*	4023.80	1106.10	227.00	234.80	58.20	71.22*	19.58*	4.02	4.16*	1.03
Hanjiang	23.44	116.88	Asia	river	0.51*	0.53*	-0.15	-0.25	116.81	10.06	43.39	1.04	17.84	61.76*	5.32	22.94	0.55	9.43
Hooghly	21.95	88.05	Asia	wave	0.19	0.26	0.03	0.07	35.53	157.72	33.29	0.00	49.12	12.89	57.22	12.08	0.00	17.82
Huanghe	37.74	118.77	Asia	river	0.65*	0.63*	0.53*	0.32	1141.43	126.63	406.53	0.09	65.03	65.61*	7.28	23.37*	0.01	3.74
Irrawaddy	18.09	95.42	Asia	tide	0.12	0.43*	0.02	0.12	14.84	20.80	0.38	0.03	4.77	36.35	50.97*	0.92	0.07	11.69
Kajiayanhe	18.41	121.62	Asia	wave	0.35	0.36	-0.21	0.22	0.46	51.39	17.22	21.98	19.57	0.41	46.46	15.57	19.87	17.69
Kynon	66.21	43.69	Europe	tide	-0.40*	-0.36	0.35	-0.19	377.41	261.37	0.01	204.86	91.44	40.36*	27.95	0.00	21.91	9.78
La Dordogne	45.6	-1.14	Europe	tide	0.26	0.37*	-0.30	-0.53*	2105.24	610.79	530.05	511.77	123.04	54.25	15.74*	13.66	13.19*	3.17

Larang	2.11	111.18	Asia	river	-0.57*	-0.59*	-0.34	0.60*	11.73	56.23	17.94	25.35	6.83	9.93	47.62*	15.20	21.47*	5.79
Le Congo	-6.02	12.29	Africa	river	0.03	0.11	-0.37*	0.21	726.87	9.17	163.94	454.33	145.07	48.48	0.61	10.93	30.30	9.68
Limpopo	-25.18	33.52	Africa	tide	0.75*	0.76*	-0.28	0.01	22.89	900.24	37.29	140.34	28.88	2.03	79.69*	3.30	12.42	2.56
Loire	47.18	-2.33	Europe	tide	0.50*	0.54*	-0.43*	0.29	6.26	166.25	0.38	57.78	20.68	2.49	66.14*	0.15	22.99	8.23
Mahanadi	20.45	85.67	Asia	tide	0.06	0.06	0.21	0.20	0.86	0.04	15.58	16.99	22.24	1.55	0.07	27.97	30.50	39.92
Mahi	22.22	72.56	Asia	river	-0.07	-0.06	-0.49*	0.19	919.85	71.41	569.82	545.58	808.48	31.55	2.45	19.55	18.72	27.73
Mangoky	-21.41	43.43	Africa	wave	0.27	0.32	-0.21	0.24	66.51	50.27	16.18	29.19	20.19	36.48	27.57	8.87	16.01	11.07
Mekong	10.93	105.18	Asia	tide	0.18	0.18	-0.04	0.21	0.16	293.12	411.92	22.66	499.94	0.01	23.87	33.55	1.85	40.72
Minjiang	26.09	119.67	Asia	wave	0.24	0.20	0.35	0.14	180.09	50.77	31.03	241.64	66.99	31.57	8.90	5.44	42.36	11.74
Narmada	21.62	72.58	Asia	tide	-0.23	-0.23	0.30	-0.11	13.95	437.45	89.24	849.56	413.75	0.77	24.25	4.95	47.09	22.94
Niger	5.57	6.58	Africa	river	0.53*	0.51*	0.06	-0.12	9.22	174.94	48.57	27.57	25.08	3.23	61.30*	17.02	9.66	8.79
Nile	30.14	31.18	Africa	wave	0.32	0.27	-0.05	0.27	1.54	0.16	0.93	0.31	0.75	41.71	4.26	25.27	8.34	20.41
Ohra	63.96	37.95	Europe	river	-0.24	-0.13	0.61*	-0.05	750.09	2716.02	26.73	1942.31	408.22	12.84	46.48	0.46	33.24	6.99
Onehek	73.15	119.48	Asia	river	0.04	-0.02	0.09	-0.03	38.26	105.73	3.49	106.30	247.84	7.63	21.08	0.70	21.19	49.41
Orange	-28.62	16.46	Africa	tide	0.72*	0.63*	-0.16	-0.08	335.41	14.09	1.22	7.61	18.47	89.02*	3.74	0.32	2.02	4.90
Pearl	22.73	113.64	Asia	river	0.54*	0.56*	-0.09	-0.05	82.62	16.34	6.37	1.41	10.97	70.18*	13.88	5.41	1.20	9.32
Peka Tynoma	69.36	33.56	Europe	wave	-0.12	-0.22	0.44*	0.19	6.03	32.72	15.11	14.92	23.93	6.50	35.30	16.30	16.09	25.81
Pinega	64.67	39.79	Europe	river	0.14	0.15	-0.52*	-0.05	48.86	7.32	4.57	198.56	135.57	12.37	1.85	1.16	50.28	34.33
Po	44.98	12.04	Europe	river	0.52*	0.51*	0.14	0.15	45.25	1.28	0.19	0.00	7.67	83.20*	2.34	0.35	0.00	14.11
Podolian Upland	46.62	31.95	Europe	river	0.22	0.22	0.25	0.17	2.71	0.03	0.58	1.60	3.11	33.74	0.41	7.27	19.92	38.66
Puer	67.61	77.8	Asia	river	-0.23	-0.28	0.53*	-0.19	744.50	97.50	331.30	3715.50	268.90	14.43	1.89	6.42	72.04	5.21
Pungwe	-19.83	34.8	Africa	wave	0.21	0.24	0.34	0.29	53.19	943.69	949.75	1840.31	452.52	1.25	22.26	22.40	43.41	10.67
Qiantang	30.31	120.96	Asia	river	-0.48*	-0.47*	-0.07	0.34	1299.14	96.64	1326.35	17.78	186.21	44.40*	3.30	45.33	0.61	6.36
Rhone	43.33	5.08	Europe	river	0.56*	0.56*	0.11	0.29	45.00	0.06	7.21	0.59	5.58	77.02*	0.10	12.33	1.00	9.54
Rio Guadiana	37.14	-7.39	Europe	tide	0.75*	0.75*	-0.37*	0.14	58.93	0.48	12.81	1.73	1.90	77.69*	0.64	16.89*	2.28	2.50

Rio Mino	41.84	-8.91	Europe	wave	0.20	0.34	-0.14	0.48*	1.54	13.15	2.93	0.79	1.25	7.84	66.87	14.88	4.04	6.36
River Gambie	13.54	-16.62	Africa	river	0.69*	0.7*	-0.32	-0.58*	97.80	5.61	16.54	0.06	5.44	77.96*	4.47	13.18	0.05	4.34
Salween	16.46	97.39	Asia	river	-0.16	-0.11	0.29	-0.01	167.57	301.99	101.75	397.09	344.72	12.76	23.00	7.75	30.24	26.25
Seine	49.43	0.09	Europe	wave	0.21	0.21	0.51*	-0.41*	5.49	0.73	35.02	8.94	4.94	9.96	1.32	63.54*	16.21	8.96
Senegal	16	-16.54	Africa	wave	-0.03	-0.03	-0.26	0.07	0.20	0.23	5.26	15.25	15.41	0.55	0.63	14.48	41.95	42.39
Sittaung	17.1	96.94	Asia	tide	0.01	-0.14	0.03	0.30	2.23	354.72	1025.32	84.18	768.82	0.10	15.87	45.87	3.77	34.39
St George	44.92	29.55	Europe	river	0.21	0.19	-0.10	0.28	7.05	4.72	11.62	6.27	8.53	18.45	12.37	30.43	16.41	22.33
Sungai	1.85	100.95	Asia	wave	-0.18	-0.30	-0.46*	-0.27	316.80	1028.18	384.52	510.33	185.14	13.06	42.40	15.86	21.04	7.63
Tanmbipa	75.89	99.75	Asia	wave	0.27	0.24	0.45*	-0.13	309.04	48.36	39.38	702.33	191.37	23.95	3.75	3.05	54.42	14.83
Tigris	30.49	47.87	Asia	river	-0.29	-0.19	0.60*	-0.53*	861.42	827.77	2010.20	152.71	486.87	19.85	19.08	46.33*	3.52	11.22
Unknown	34.28	-6.69	Africa	wave	0.77*	0.75*	0.14	0.27	304.19	70.53	9.29	1.23	7.99	77.36*	17.94*	2.36	0.31	2.03
Unkonwn	58.19	-67.66	Europe	tide	-0.37*	-0.35	0.26	0.23	2911.68	37.01	1105.74	667.94	1030.78	50.61	0.64	19.22	11.61	17.92
Weser	53.55	8.55	Europe	wave	0.42*	0.21	0.02	0.36	184.12	223.61	24.52	37.77	36.40	36.36*	44.15	4.84	7.46	7.19
Xatanhra	72.91	105.93	Asia	river	-0.08	-0.07	-0.29	-0.32	12.15	193.81	849.63	99.94	57.33	1.00	15.98	70.05	8.24	4.73
Yalu	39.85	124.31	Asia	tide	-0.43*	-0.36	-0.38*	0.64*	283.96	184.99	217.76	56.89	47.92	35.88*	23.37*	27.51*	7.19	6.05
Yangtze	31.4	121.99	Asia	river	0.77*	0.76*	0.50*	0.71*	6191.10	367.70	277.10	442.10	190.60	82.90*	4.92	3.71	5.92	2.55
Albert	-17.54	139.76	Oceania	river	-0.08	0.03	-0.35	0.33	6.47	0.70	177.80	147.25	42.58	1.73	0.19	47.44	39.29	11.36
Anadyr	64.8	176.4	Asia	river	0.13	0.18	-0.74*	0.69*	115.12	247.79	859.71	407.25	64.85	6.79	14.62	50.73*	24.03*	3.83
Digoel	-7.11	138.61	Asia	tide	-0.14	0.04	0.13	0.24	1.14	2.95	4.42	1.30	4.27	8.12	20.94	31.37	9.26	30.31
Fly	-8.47	143.45	Oceania	tide	0.54*	0.59*	-0.11	-0.40*	331.80	105.11	49.60	47.20	38.48	57.99*	18.37	8.67	8.25	6.73
Imjin	37.79	126.53	Asia	river	-0.25	-0.26	0.05	0.25	115.65	77.78	274.41	1.84	88.67	20.71	13.93	49.15	0.33	15.88
Kelyma	69.5	161.13	Asia	river	0.57*	0.29	-0.37*	-0.24	1018.05	351.01	277.18	37.39	88.50	57.45*	19.81	15.64*	2.11	4.99
Nakdonggang	35.06	128.93	Asia	river	0.84*	0.86*	-0.54*	0.17	116.11	9.17	3.43	1.47	2.09	87.78*	6.93*	2.59	1.11	1.58
Norman	-17.43	140.81	Oceania	river	0.47*	0.5*	0.10	-0.33	54.95	8.03	36.60	2.68	9.24	49.28*	7.20	32.82	2.40	8.29
Ord	-15.5	128.35	Oceania	wave	-0.35	-0.22	0.12	-0.39*	45.26	8.53	16.90	5.91	20.74	46.50	8.76	17.36	6.07	21.30

Oxota	59.27	143.1	Asia	wave	0.66*	0.65*	-0.45*	0.05	104.69	4.47	2.51	15.94	6.84	77.86*	3.32	1.87	11.86	5.09
Penzhina	62.53	164.68	Asia	tide	0.40*	0.25	0.19	0.20	67.43	13.76	26.99	15.76	18.51	47.34*	9.66	18.95	11.06	12.99
Ramu	-4	144.68	Oceania	river	0.12	-0.18	0.10	0.29	0.87	3.02	1.71	0.98	3.24	8.88	30.72	17.41	10.01	32.98
Sepik	-3.81	144.56	Oceania	river	-0.03	-0.16	0.06	-0.43*	0.09	45.69	92.20	2.54	31.97	0.05	26.49	53.45	1.47	18.53
Tuman	42.28	130.72	Asia	wave	0.16	0.15	-0.14	-0.46*	21.08	7.95	63.70	6.87	14.46	18.48	6.97	55.85	6.02	12.68
Unknown	59.62	149.12	Asia	wave	0.67*	0.67*	0.08	-0.02	500.16	0.56	20.87	10.04	36.99	87.96*	0.10	3.67	1.77	6.51
Unknown	66.14	44.05	Asia	wave	-0.22	-0.21	-0.17	0.01	9.46	0.52	0.00	5.95	11.71	34.23	1.89	0.00	21.53	42.36
Unknown	68.91	170.43	Asia	River	0.26	0.21	0.49*	0.05	89.96	225.77	0.12	126.19	58.83	17.96	45.08	0.02	25.19	11.75
Victoria	-14.89	129.52	Oceania	tide	0.14	0.01	-0.19	0.00	261.07	221.67	268.03	574.20	694.26	12.93	10.98	13.27	28.44	34.38
Albany	52.28	-81.41	North America	river	0.27	0.25	-0.14	-0.12	769.14	1039.67	202.78	353.12	497.29	26.87	36.33	7.09	12.34	17.38
Altamaha	31.31	-81.26	North America	tide	0.67*	0.63*	-0.22	-0.02	699.60	185.03	0.18	23.71	39.59	73.79*	19.52*	0.02	2.50	4.18
Amazon	0.45	-49.81	South America	tide	0.74*	0.73*	0.06	0.22	5357.60	32.60	41.80	12.50	266.60	93.81*	0.57	0.73	0.22	4.67
Apalachicola	29.72	-84.96	North America	wave	0.65*	0.42*	-0.37*	0.32	439.81	29.47	5.37	53.64	33.01	78.36*	5.25	0.96	9.56	5.88
Attawapiskat	52.98	-82.21	North America	wave	0.40*	0.13	-0.10	0.12	3888.40	1003.50	200.10	150.50	1202.10	60.34*	15.57	3.10	2.34	18.65
Brazos	28.89	-95.39	North America	river	0.71*	0.81*	-0.33	-0.04	830.30	269.53	15.20	20.34	32.51	71.09*	23.08*	1.30	1.74	2.78
Cape Fear	33.86	-78.04	North America	tide	0.25	0.34	-0.38*	-0.02	17.37	16.93	0.00	45.85	12.86	18.67	18.20	0.00	49.29	13.83
Chowan	35.99	-76.64	North America	river	0.50*	0.42*	-0.24	0.14	2534.49	164.82	214.92	274.13	442.49	69.80*	4.54	5.92	7.55	12.19
Connecticut	41.25	-72.33	North America	wave	0.34	0.15	-0.14	0.51*	1.70	0.28	2.34	0.18	0.61	33.32	5.45	45.80	3.42	12.00
Cote Node	50.11	-61.83	North America	wave	0.16	0.15	-0.13	-0.01	1.31	1.00	0.75	0.55	2.82	20.43	15.58	11.64	8.49	43.87
Courantyne	6.2	-57.05	South America	wave	0.14	0.21	0.10	0.36	115.16	1984.54	26.65	5.33	226.70	4.88	84.15	1.13	0.23	9.61
Delaware	39.31	-75.39	North America	tide	-0.02	-0.10	0.41*	0.42*	0.00	11.54	41.86	26.59	10.11	0.00	12.81	46.46*	29.51	11.22
Essequibo	7.06	-58.34	South America	river	0.65*	0.65*	-0.57*	0.53*	4402.50	3.50	98.00	28.10	374.40	89.73*	0.07	2.00	0.57	7.63
Fuerte	25.76	-109.4	North America	wave	0.16	0.17	0.16	-0.06	3.27	3.05	0.23	3.87	7.75	18.01	16.79	1.25	21.31	42.63
Harricana	51.2	-79.81	North America	wave	0.66*	0.68*	-0.42*	-0.11	29739.20	3150.10	1545.00	10111.50	1499.20	64.59*	6.84	3.36	21.96	3.26
Hayes	57.07	-92.15	North America	wave	0.26	0.29	0.02	-0.29	230.13	76.77	285.52	0.06	170.45	30.16	10.06	37.42	0.01	22.34

James	37.07	-76.6	North America	river	0.64*	0.66*	0.03	0.26	339.33	31.53	0.00	25.33	26.78	80.23*	7.45	0.00	5.99	6.33
Unknown	52.23	-78.61	North America	river	-0.02	0.01	0.11	-0.06	5.90	2673.14	46.61	15.01	521.68	0.18	81.94	1.43	0.46	15.99
Jequitinhonha	-15.85	-38.83	South America	wave	0.55*	0.38*	-0.54*	0.47*	149.63	51.07	48.74	108.62	7.99	40.88*	13.95*	13.31*	29.67*	2.18
Kitikmeot	66.86	-108.1	North America	wave	0.42*	0.27	0.06	-0.12	513.27	0.45	2.55	132.43	137.50	65.28*	0.06	0.32	16.84	17.49
Koksoak	58.56	-68.15	North America	tide	-0.05	0.08	0.64*	-0.05	0.09	0.78	0.10	20.80	1.58	0.38	3.35	0.45	89.05	6.78
Kuskokwim	59.84	-162.4	North America	river	0.25	0.38*	0.40*	0.59*	6.09	147.81	137.77	57.69	16.13	1.67	40.44*	37.69*	15.79*	4.41
Machenzie	69.36	-133.9	North America	river	-0.09	-0.06	0.14	0.06	118.70	2.15	20.97	109.37	355.64	19.56	0.35	3.46	18.02	58.61
Magdalena	11.01	-74.78	South America	tide	-0.26	-0.29	-0.30	-0.15	20.09	3.96	2.58	82.75	11.31	16.65	3.28	2.14	68.56	9.37
Maroni	5.82	-53.95	South America	wave	0.69*	0.73*	-0.02	0.71*	800.94	244.84	67.92	7.30	33.71	69.36*	21.20*	5.88	0.63	2.92
Mississippi	29.62	-89.9	North America	river	0.44*	0.15	-0.19	0.13	211.59	89.95	5.28	0.64	47.57	59.60*	25.34	1.49	0.18	13.40
Moisie	50.18	-66.06	North America	wave	-0.39*	-0.43*	-0.38*	0.17	17.76	6.32	0.17	5.43	5.29	50.78*	18.08	0.48	15.52	15.13
Moose	51.39	-80.36	North America	wave	0.42*	0.41*	-0.24	-0.18	6552.80	392.30	67.50	9284.10	1349.70	37.13*	2.22	0.38	52.61	7.65
Nelson	57.06	-92.53	North America	river	0.78*	0.78*	0.13	-0.34	453.94	31.41	7.12	28.48	13.64	84.91*	5.88	1.33	5.33	2.55
Norddu Quebec	53.85	-79.11	North America	river	-0.10	-0.08	0.02	-0.03	2.12	5.62	0.00	0.01	13.46	9.98	26.51	0.01	0.06	63.43
Rio de la Plata	-33.72	-59.29	South America	river	-0.25	-0.21	-0.32	0.41*	1048.50	81.70	3295.20	3255.60	750.50	12.44	0.97	39.08	38.61*	8.90
Potomac	38	-76.35	North America	river	0.71*	0.7*	0.17	0.15	83.72	7.78	0.34	3.26	4.51	84.05*	7.81	0.34	3.27	4.53
Ribeira de Iguape	-24.7	-47.41	South America	wave	0.30	0.28	-0.16	0.24	11.93	0.17	15.02	0.89	6.54	34.55	0.48	43.47	2.58	18.92
Rio Biobio	-36.83	-73.21	South America	wave	0.27	0.27	0.09	0.19	4.03	0.03	3.04	2.09	2.90	33.29	0.21	25.16	17.31	24.03
Riode Contas	-14.26	-38.97	South America	wave	0.55*	0.55*	-0.11	0.32	5.18	0.05	1.76	0.17	0.62	66.52*	0.70	22.63	2.17	7.98
Rio Doce	-19.63	-39.78	South America	wave	0.22	0.23	-0.40*	0.01	13.41	0.10	1.25	0.01	15.75	43.94	0.33	4.11	0.03	51.60
Rio Grijalva	18.61	-92.7	North America	river	0.00	-0.01	-0.08	0.02	0.00	0.03	0.06	0.70	6.37	0.02	0.46	0.80	9.82	88.91
Rio Guama	-1.16	-48.51	South America	tide	0.39*	0.18	0.41*	0.01	149.64	28.85	18.20	124.79	40.48	41.34*	7.97	5.03	34.48	11.18
Rio Guayas	-2.7	-79.92	South America	tide	0.33	0.46*	-0.20	-0.25	0.18	25.08	0.35	1.59	5.55	0.54	76.58*	1.06	4.87	16.96
Rio Lempa	13.23	-88.84	South America	wave	0.05	0.01	0.66*	0.22	53.53	0.02	0.69	0.94	8.36	84.25	0.03	1.08	1.47	13.16
Rio Maule	-35.3	-72.43	South America	wave	0.50*	0.46*	-0.51*	-0.30	15.33	12.50	0.37	3.82	1.76	45.38*	37.00*	1.11	11.31	5.20

Rio Mearim	-2.57	-44.45	South America	tide	-0.25	-0.29	-0.51*	0.25	7.60	241.85	130.67	391.40	84.41	0.89	28.26	15.27	45.73	9.86
Rio Negro	-41.07	-62.78	South America	tide	0.71*	0.54*	0.35	-0.15	130.95	171.74	0.14	24.56	16.59	38.07*	49.93*	0.04	7.14	4.82
Rio Paraiba do Sul	-21.61	-40.99	South America	wave	0.75*	0.75*	-0.14	0.37*	103.88	2.44	3.79	2.26	4.44	88.94*	2.09	3.24	1.94	3.80
Rio Parnaiba	-2.71	-41.78	South America	river	0.64*	0.64*	-0.24	-0.30	196.99	17.58	11.28	0.22	15.48	81.56*	7.28	4.67	0.09	6.41
Rio Patia	2.63	-78.41	South America	wave	0.18	0.28	0.55*	-0.28	6.48	48.98	0.40	22.35	7.40	7.57	57.21	0.47	26.11	8.64
Riviere Manicouagan	49.17	-68.21	North America	river	-0.33	-0.27	0.13	-0.25	182.48	194.69	0.66	52.29	75.04	36.12	38.54	0.13	10.35	14.85
Riviere Saguenay	48.1	-69.68	North America	tide	0.35	0.15	0.45*	-0.44*	2.97	1.16	4.99	3.14	0.87	22.61	8.85	38.02*	23.92*	6.60
Rupert	51.47	-78.84	North America	river	-0.01	0.06	-0.42*	0.08	77.60	20682.30	255.40	441.80	980.70	0.35	92.18	1.14	1.97	4.37
Saint Lawrence	47.1	-70.6	North America	tide	0.22	0.15	-0.27	0.05	188.95	31.58	5.32	136.10	98.50	41.04	6.86	1.16	29.56	21.39
Severn	56.13	-87.53	North America	wave	0.27	0.27	-0.37*	0.35	72.61	69.99	154.79	183.30	50.34	13.67	13.18	29.15*	34.52	9.48
St John	45.2	-66.1	North America	wave	-0.12	-0.23	0.18	0.14	1.48	11.32	1.40	2.18	3.45	7.44	57.11	7.05	10.98	17.41
Susquehanna	39.21	-76.34	North America	river	0.54*	0.46*	0.00	0.36	131.89	0.42	27.44	13.54	17.75	69.04*	0.22	14.36	7.09	9.29
Tensaw	30.64	-87.98	North America	river	0.59*	0.44*	0.14	0.08	420.92	2.08	20.97	6.57	48.53	84.34*	0.42	4.20	1.32	9.72
Ungava Bay	59.99	-69.65	North America	tide	0.14	0.21	0.58*	-0.12	0.69	5.21	0.58	10.80	1.43	3.68	27.87	3.10	57.71	7.65
Unknown	52.93	141.15	North America	tide	0.39*	0.39*	0.38*	-0.50*	6.81	0.01	7.38	8.53	1.41	28.20*	0.02	30.59*	35.35*	5.84
Unknown	59.31	144.92	North America	tide	-0.01	0.01	0.51*	-0.03	0.00	0.44	0.03	8.43	1.51	0.02	4.25	0.25	80.98	14.51
Winisk	55.32	-85.06	North America	wave	0.53*	0.22	-0.44*	0.06	947.85	218.55	114.01	212.22	119.34	58.80*	13.56	7.07	13.17	7.40
Wood	58.79	-158.7	North America	wave	-0.27	-0.19	0.10	0.07	75.55	289.59	6.25	4.52	42.16	18.07	69.27	1.50	1.08	10.08
Yukon	62.56	-165.1	North America	wave	0.29	0.10	0.13	-0.06	142.72	142.72	22.93	20.03	92.94	33.87	33.87	5.44	4.75	22.06

**P*<0.05; Mean Sq: Mean square

Supplementary Notes

Supplementary Note 1: Accuracy assessments for typical existing suspended sediment concentration (SSC) inversion algorithms

Over the past decades, near-surface SSC retrievals in site-specific studies often relied on a single red band (Miller and McKee 2004; Petus et al. 2014; Shi et al. 2015), which typically demonstrated accurate estimates within relatively clear waters (e.g., SSC < 100 mg/L). Nevertheless, in extremely turbid conditions (e.g., SSC >1000 mg/L), the red band may become insensitive to SSC and may even exhibit a saturation effect (Luo et al. 2018; Shen et al. 2010; Shi et al. 2018; Wei et al. 2021; Yu et al. 2019). In such cases, combining the red band with the near-infrared (NIR) band or even shortwave infrared (SWIR) band has been a common approach for coastal SSC retrieval by many studies (Balasubramanian et al. 2020; Doxaran et al. 2002; He et al. 2013; Zhang et al. 2010). However, most existing algorithms were developed for a specific location with limited range of SSCs, which limits their applicability in large-scale areas where SSCs can vary over several orders of magnitude. To address these problems, many algorithms employed a blending scheme to smooth the transitions between different SSC algorithm outputs, enabling accurate SSC inversions from clear to turbid water (Dogliotti et al. 2015; Feng et al. 2014; Han et al. 2016; Novoa et al. 2017). Nevertheless, most of these algorithms require reflectance thresholds from certain wavelengths as blending boundaries, which vary across different studies. Recently, numerous algorithms have been developed to seamlessly retrieve coastal SSC across clear to turbid conditions (Balasubramanian et al. 2020; Wei et al. 2021; Yu et al. 2019), enabling the quantification of coastal sediment at a global scale.

Herein, we initially conducted accuracy assessments for certain of these algorithms to select the most accurate one for SSC inversion in this study. These include the algorithm from Han et al. (2016) (hereafter as Han_adapted), Feng et al. (2014) (hereafter as Feng_adapted), and Yu et al. (2019) (hereafter as Yu_adapted). Note that we did not evaluate the global coastal SSC algorithm from Wei et al. (2021), primarily because it is an improvement of the algorithm in Yu et al. (2019), which adds two additional blue bands based on the algorithm in Yu et al. (2019) to improve the SSC inversion accuracy in clear water. However, these two blue bands are not available in MODIS surface reflectance (SR) products. Additionally, we did not assess the global algorithm developed by Balasubramanian et al. (2020), mainly because this algorithm heavily depends on one of the inherent optical properties (IOP)-particulate backscattering, which cannot be directly derived from the MODIS SR data.

The reflectance data utilized in Han et al. (2016), Feng et al. (2014), and Yu et al. (2019) were all remote sensing reflectance (Rrs), generated using an atmospheric correction approach tailored for ocean color application (Wang and Shi 2007). This Rrs differs from MODIS SR, which was generated using a land-based atmospheric correction algorithm. Furthermore, the algorithm in Yu et al. (2019) was primarily designed for VIIRS satellite data, meaning its spectral response function for certain bands differs from that of MODIS. Consequently, we initially used our collected in-situ measured SSC and the corresponding daily MODIS Aqua SR data to recalibrate the algorithm parameters for these three algorithms. Subsequently, we assessed the accuracy of these algorithms. The processing details are outlined below:

Han_adapted. We utilized the red (645 nm) and NIR (859 nm) bands from MODIS SR products to replace the red (671 nm) and NIR (745 nm) bands in Han et al. (2016) for SSC retrieval. In the adapted algorithm, we employed two thresholds (0.04 and 0.09) based on the red band reflectance (R_{645}) as the blending boundaries. These two thresholds were determined based on the in-situ measured SSC and the corresponding red reflectance from MODIS Aqua SR. Therefore, the algorithm was defined as follows:

$$\mathrm{SSC_{clear}} = A_T(\lambda_1)\rho_W(\lambda_1)/(1 - \rho_W(\lambda_1)/C(\lambda_1)) \quad (1)$$

$$\mathrm{SSC_{turbid}} = A_T(\lambda_2)\rho_W(\lambda_2)/(1 - \rho_W(\lambda_2)/C(\lambda_2)) \quad (2)$$

$$\mathrm{SSC_{final}} = \frac{W_c * \mathrm{SSC}_{clear} + W_t * \mathrm{SSC}_{turbid}}{W_c + W_t} \quad (3)$$

$$W_c = \log_{10} 0.04 - \log_{10}(R_{645}) \quad (4)$$

$$W_t = \log_{10}(R_{645}) - \log_{10} 0.03 \quad (5)$$

Where $A_T(\lambda)$ and $C(\lambda)$ are wavelength-dependent coefficients, with λ_1 and λ_2 are set to 645 and 859 nm, respectively. The values of $A_T(\lambda)$ and $C(\lambda)$ for λ_1 are 228.1 and 0.164, and for λ_2 are 3078.9 and 0.211. These coefficients are obtained from Dogliotti et al. (2015). The parameters W_c and W_t represent the weights for the SSC estimations from clear to turbid waters, respectively. These weights are determined based on the value of R_{645}. Specifically, if R_{645} <0.04, W_c is 1 and W_t is 0. Conversely, if R_{645} >0.09, W_c is 0 and W_t is 1. For R_{645} values between these two boundaries, W_c and W_t are calculated using the formula above.

Feng_adapted. We replaced the single band reflectance (645 nm) in Feng et al. (2014) with a band ratio (R_{645}/R_{555}, which is the ratio of SR between MODIS 645 and 555 nm bands) for sediment retrieval in relatively clear waters. Additionally, we utilized the ratio between 859 and 645 nm (R_{859}/R_{645}) for sediment retrieval in highly turbid waters. The algorithm was defined as follows:

$$\mathrm{SSC_{Low}}=0.82*e^{4.62*(R645/R555)} \quad (6)$$

$$\mathrm{SSC_{High}}=336.23*(R_{859}/R_{645})^2+387.58*(R_{859}/R_{645}) \quad (7)$$

$$\mathrm{SSC_{Middle}}=\alpha*\mathrm{SSC_{low}}+\beta*\mathrm{SSC_{high}} \quad (8)$$

$$\mathrm{SSC(mg/L)} = \begin{cases} \mathrm{SSC_{Low}}\ (\mathrm{SSC_{Low}} < 50\ \mathrm{mg/L}) \\ \mathrm{SSC_{High}}\ (150\ \mathrm{mg/L} < \mathrm{SSC_{Low}}) \\ \mathrm{SSC_{Middle}}\ (50\ \mathrm{mg/L} \le \mathrm{SSC_{Low}} \le 150\ \mathrm{mg/L}) \end{cases}$$

Similar to Feng et al. (2014), we defined the α as $\ln(150/SSC_{Low})/\ln(150/50)$, and β as $\ln(SSC_{Low}/50)/\ln(150/50)$. In this adapted algorithm, the SSC_{Low} was first calculated using R_{645}/R_{555}, and then the retrieval algorithm was determined based on SSC_{Low}. For the relatively clear water (SSC<50 mg/L), SSC_{Low} was adopted, and for water with SSC>150 mg/L, the SSC_{High} was selected. For the intermediate SSC values, a mixture of SSC_{Low} and SSC_{High} was applied to remove the discontinuity between these two algorithms.

Yu_adapted. The blue (R_{469}), green (R_{555}), red (R_{645}), and NIR (R_{859}) bands from MODIS SR products replaced the bands at 486, 551, 671, and 862 nm utilized in Yu et al. (2019) for SSC inversion. A band of 745 nm used in Yu et al. (2019), which was manually aggregated, was not used

in this study. We calibrate the parameter utilized in Yu et al. (2019) using the least squares error minimization principle, resulting in the algorithm as follows:

$$SSC=exp(0.859*(0.145*(R_{555}/R_{469})+(5.167*(R_{645}/R_{555})*(R_{645}/(R_{645}+R_{859}))+(7.244*(R_{859}/R_{555})*(R_{859}/(R_{645}+R_{859}))))\text{^}0.990) \quad (9)$$

Subsequently, we compared the retrieved SSC from these three adapted algorithms mentioned above with in-situ measured SSC. We found that the Yu_adapted algorithm demonstrated the highest accuracy among the three algorithms (Supplementary Table 1). Consequently, the Yu_adapted algorithm was chosen for SSC inversion in this research.

However, we noted significant uncertainty in the Yu_adapted algorithm when applied to turbid water bodies (i.e., >500 mg/L), possibly due to the limited number of in-situ samples from turbid water bodies. Despite collecting a large amount of in-situ SSC data, interference from clouds and coarse spatial resolution of MODIS led to the land adjacency effect, resulting in only a relatively small number of in-situ data being matched with satellite observations. Given the long-term average coastal SSC of the majority of deltas globally does not exceed 500 mg/L (Wei et al. 2021), to mitigate the impact of high-turbidity water bodies on the algorithm, we assumed that SSC inversed by the Yu_adapted algorithm exceeding 500 mg/L would likely have considerable errors and thus should be excluded. Therefore, only the inverted SSC lower than 500 mg/L was considered reliable and used for further analysis. We chose 500 mg/L instead of 100 mg/L because while the monthly mean SSC in most deltas is likely below 100 mg/L, some deltas may indeed experience high SSC at times. Limiting SSC to 100 mg/L would therefore result in a lack of data for many of these deltas.

References

1. Azhikodan, G., & Yokoyama, K.J.C.S.R. (2016). Spatio-temporal variability of phytoplankton (Chlorophyll-a) in relation to salinity, suspended sediment concentration, and light intensity in a macrotidal estuary. *Continental Shelf Research*, 126, 15-26
2. Balasubramanian, S.V., Pahlevan, N., Smith, B., Binding, C., Schalles, J., Loisel, H., Gurlin, D., Greb, S., Alikas, K., & Randla, M. (2020). Robust algorithm for estimating total suspended solids (TSS) in inland and nearshore coastal waters. *Remote Sensing of Environment, 246*, 111768
3. Cheevaporn, V., & Menasveta, P. (2003). Water pollution and habitat degradation in the Gulf of Thailand. *Marine Pollution Bulletin*, 47, 43-51
4. Chen, J., D'Sa, E., Cui, T., & Zhang, X.J.O.E. (2013). A semi-analytical total suspended sediment retrieval model in turbid coastal waters: A case study in Changjiang River Estuary. *Optics Express*, 21, 13018-13031
5. Chen, S., Huang, W., Chen, W., & Wang, H.J.E.I. (2011). Remote sensing analysis of rainstorm effects on sediment concentrations in Apalachicola Bay, USA. *Ecological infromatics*, 6, 147-155
6. Dalai, T.K., Nishimura, K., & Nozaki, Y. (2005). Geochemistry of molybdenum in the Chao Phraya River estuary, Thailand: Role of suboxic diagenesis and porewater transport. *Chemical Geology*, 218, 189-202
7. Dogliotti, A.I., Ruddick, K., Nechad, B., Doxaran, D., & Knaeps, E. (2015). A single algorithm to retrieve turbidity from remotely-sensed data in all coastal and estuarine waters. *Remote Sensing of Environment, 156*, 157-168
8. Doxaran, D., Froidefond, J.-M., Lavender, S., & Castaing, P. (2002). Spectral signature of highly turbid waters Application with SPOT data to quantify suspended particulate matter concentrations. *Remote Sensing of Environment, 81*, 149-161
9. Falcini, F., Khan, N.S., Macelloni, L., Horton, B.P., Lutken, C.B., McKee, K.L., Santoleri, R., Colella, S., Li, C., & Volpe, G. (2012). Linking the historic 2011 Mississippi River flood to coastal wetland sedimentation. *Nature Geoscience*, 5, 803-807
10. Feng, L., Hu, C., Chen, X., & Song, Q. (2014). Influence of the Three Gorges Dam on total suspended matters in the Yangtze Estuary and its adjacent coastal waters: Observations from MODIS. *Remote Sensing of Environment, 140*, 779-788
11. Flores, R.P., Rijnsburger, S., Horner-Devine, A.R., Souza, A.J., & Pietrzak, J.D. (2017). The impact of storms and stratification on sediment transport in the R hine region of freshwater influence. *Journal of Geophysical Research: Oceans*, 122, 4456-4477
12. Gallay, M., Martinez, J.-M., Mora, A., Castellano, B., Yépez, S., Cochonneau, G., Alfonso, J.A., Carrera, J.M., López, J.L., & Laraque, A.J.J.o.S.A.E.S. (2019). Assessing Orinoco river sediment discharge trend using MODIS satellite images. *Journal of South American Earth Sciences*, 91, 320-331
13. Gensac, E., Martinez, J.-M., Vantrepotte, V., & Anthony, E. (2016). Seasonal and inter-annual dynamics of suspended sediment at the mouth of the Amazon river: The role of continental and oceanic forcing, and implications for coastal geomorphology and mud bank formation. *Continental Shelf Research*, 118, 49-62
14. Han, B., Loisel, H., Vantrepotte, V., Mériaux, X., Bryère, P., Ouillon, S., Dessailly, D., Xing, Q., & Zhu, J. (2016). Development of a Semi-Analytical Algorithm for the Retrieval of

Suspended Particulate Matter from Remote Sensing over Clear to Very Turbid Waters. *Remote Sensing, 8(211)*

15. He, X., Bai, Y., Pan, D., Huang, N., Dong, X., Chen, J., Chen, C.-T.A., & Cui, Q. (2013). Using geostationary satellite ocean color data to map the diurnal dynamics of suspended particulate matter in coastal waters. *Remote Sensing of Environment, 133*, 225-239
16. Islam, M.R., Begum, S.F., Yamaguchi, Y., & Ogawa, K. (2002). Distribution of suspended sediment in the coastal sea off the Ganges–Brahmaputra River mouth: observation from TM data. *Journal of Marine systems*, 32, 307-321
17. Jayaram, C., Patidar, G., Swain, D., Chowdary, V., & Bandyopadhyay, S. (2021). Total Suspended Matter Distribution in the Hooghly River Estuary and the Sundarbans: A Remote Sensing Approach. *IEEE Journal of Selected Topics in Applied Earth Observations Remote Sensing*, 14, 9064-9070
18. Kostaschuk, R., Stephan, B., & Luternauer, J. (1993). Suspended sediment concentration in a buoyant plume: Fraser River, *Canada. Geo-Marine Letters*, 13, 165-171
19. Lahet, F., Ouillon, S., & Forget, P. (2000). A three-component model of ocean color and its application in the Ebro River mouth area. *Remote Sensing of Environment*, 72, 181-190
20. Lahet, F., Ouillon, S., & Forget, P. (2001). Colour classification of coastal waters of the Ebro river plume from spectral reflectances. *International Journal of Remote Sensing*, 22, 1639-1664
21. Lorthiois, T., Doxaran, D., & Chami, M. (2012). Daily and seasonal dynamics of suspended particles in the Rhône River plume based on remote sensing and field optical measurements. *Geo-Marine Letters*, 32, 89-101
22. Luo, Y., Doxaran, D., Ruddick, K., Shen, F., Gentili, B., Yan, L., & Huang, H. (2018). Saturation of water reflectance in extremely turbid media based on field measurements, satellite data and bio-optical modelling. *Optics Express, 26*, 10435-10451
23. Miller, R.L., & McKee, B.A. (2004). Using MODIS Terra 250 m imagery to map concentrations of total suspended matter in coastal waters. *Remote Sensing of Environment, 93*, 259-266
24. Mitra, A., & Kumar, V.S. (2021). A numerical investigation on the tide-induced residence time and its association with the suspended sediment concentration in Gulf of Khambhat, northern Arabian Sea. *Marine Pollution Bulletin*, 163, 111947
25. Mize, S.V., Murphy, J.C., Diehl, T.H., & Demcheck, D.K.J.J.o.H. (2018). Suspended-sediment concentrations and loads in the lower Mississippi and Atchafalaya rivers decreased by half between 1980 and 2015. *Journal of Hydrology*, 564, 1-11
26. Moreira, D., Simionato, C.G., Gohin, F., Cayocca, F., & Tejedor, M.L.C. (2013). Suspended matter mean distribution and seasonal cycle in the Río de La Plata estuary and the adjacent shelf from ocean color satellite (MODIS) and in-situ observations. *Continental Shelf Research*, 68, 51-66
27. Nicolini, M., Pranzini, E., & Santini, C. (1999). Landsat TM images as sea-truth data for calibrating dispersion coefficients in a two-dimensional river plume numerical model. In, *Remote Sensing for Earth Science, Ocean, and Sea Ice Applications* (pp. 590-597): International Society for Optics and Photonics
28. Novoa, S., Doxaran, D., Ody, A., Vanhellemont, Q., Lafon, V., Lubac, B., & Gernez, P. (2017). Atmospheric corrections and multi-conditional algorithm for multi-sensor remote sensing of

suspended particulate matter in low-to-high turbidity levels coastal waters. *Remote Sensing, 9*, 61

29. Oliveira, E.N.d., Knoppers, B.A., Lorenzzetti, J.A., Medeiros, P.R.P., Carneiro, M.E., & Souza, W.F.L.d. (2012). A satellite view of riverine turbidity plumes on the NE-E Brazilian coastal zone. *Brazilian Journal of Oceanography*, 60, 283-298
30. Ouillon, S., Forget, P., Froidefond, J.-M., & Naudin, J.-J. (1997). Estimating suspended matter concentrations from SPOT data and from field measurements in the Rhône river plume. *Marine Technology Society Journal*, 31, 15
31. Papenmeier, S., Schrottke, K., & Bartholomä, A. (2014). Over time and space changing characteristics of estuarine suspended particles in the German Weser and Elbe estuaries. *Journal of Sea Research*, 85, 104-115
32. Petus, C., Chust, G., Gohin, F., Doxaran, D., Froidefond, J.-M., & Sagarminaga, Y.J.C.S.R. (2010). Estimating turbidity and total suspended matter in the Adour River plume (South Bay of Biscay) using MODIS 250-m imagery. *Continental Shelf Research*, 30, 379-392
33. Petus, C., Marieu, V., Novoa, S., Chust, G., Bruneau, N., & Froidefond, J.-M. (2014). Monitoring spatio-temporal variability of the Adour River turbid plume (Bay of Biscay, France) with MODIS 250-m imagery. *Continental Shelf Research, 74*, 35-49
34. Ramaswamy, V., Rao, P., Rao, K., Thwin, S., Rao, N.S., & Raiker, V. (2004). Tidal influence on suspended sediment distribution and dispersal in the northern Andaman Sea and Gulf of Martaban. *Marine Geology*, 208, 33-42
35. Shahzad, M.I., Meraj, M., Nazeer, M., Zia, I., Inam, A., Mehmood, K., & Zafar, H. (2018). Empirical estimation of suspended solids concentration in the Indus Delta Region using Landsat-7 ETM+ imagery. *Journal of Environmental Management*, 209, 254-261
36. Shen, F., Verhoef, W., Zhou, Y., Salama, M., & Liu, X. (2010). Satellite estimates of wide-range suspended sediment concentrations in Changjiang (Yangtze) estuary using MERIS data. *Estuaries Coasts*, 33, 1420-1429
37. Shen, F., Suhyb Salama, M., Zhou, Y.-X., Li, J.-F., Su, Z., & Kuang, D.-B. (2010). Remote-sensing reflectance characteristics of highly turbid estuarine waters–a comparative experiment of the Yangtze River and the Yellow River. *International Journal of Remote Sensing, 31*, 2639-2654
38. Shi, K., Zhang, Y., Zhu, G., Liu, X., Zhou, Y., Xu, H., Qin, B., Liu, G., & Li, Y. (2015). Long-term remote monitoring of total suspended matter concentration in Lake Taihu using 250 m MODIS-Aqua data. *Remote Sensing of Environment, 164*, 43-56
39. Shi, W., Zhang, Y., & Wang, M. (2018). Deriving total suspended matter concentration from the near-infrared-based inherent optical properties over turbid waters: A case study in Lake Taihu. *Remote Sensing, 10*, 333
40. Torregroza-Espinosa, A.C., Restrepo, J.C., Correa-Metrio, A., Hoyos, N., Escobar, J., Pierini, J., & Martinez, J.-M. (2020). Fluvial and oceanographic influences on suspended sediment dispersal in the Magdalena River Estuary. *Journal of Marine systems*, 204, 103282
41. Vanhellemont, Q., Neukermans, G., & Ruddick, K. (2014). Synergy between polar-orbiting and geostationary sensors: Remote sensing of the ocean at high spatial and high temporal resolution. *Remote Sensing of Environment*, 146, 49-62
42. Wackerman, C., Hayden, A., & Jonik, J. (2017). Deriving spatial and temporal context for point measurements of suspended-sediment concentration using remote-sensing imagery in

the Mekong Delta. *Continental Shelf Research*, 147, 231-245
43. Walker, N.D. (1996). Satellite assessment of Mississippi River plume variability: causes and predictability. *Remote Sensing of Environment*, 58, 21-35
44. Wei, J., Wang, M., Jiang, L., Yu, X., Mikelsons, K., & Shen, F. (2021). Global estimation of suspended particulate matter from satellite ocean color imagery. *Journal of Geophysical Research: Oceans, 126*, e2021JC017303
45. Wolanski, E., & Gibbs, R. (1996). Flocculation of suspended sediment in the Fly River estuary, Papua New Guinea. *Oceanographic Literature Review*, 4, 357
46. Xu, J., Fang, H., Fu, S., & Huang, X. (2012). Estimating Suspended Sediment Concentrations from SPOT Image: A Case Study in Danshuihe, Taiwan. *Remote Sensing Technology Application*, 14, 17-22
47. Yu, X., Lee, Z., Shen, F., Wang, M., Wei, J., Jiang, L., & Shang, Z. (2019). An empirical algorithm to seamlessly retrieve the concentration of suspended particulate matter from water color across ocean to turbid river mouths. *Remote Sensing of Environment, 235*, 111491
48. Zhan, W., Wu, J., Wei, X., Tang, S., & Zhan, H. (2019). Spatio-temporal variation of the suspended sediment concentration in the Pearl River Estuary observed by MODIS during 2003–2015. *Continental Shelf Research*, 172, 22-32
49. Zhang, M., Dong, Q., Cui, T., Xue, C., & Zhang, S. (2014). Suspended sediment monitoring and assessment for Yellow River estuary from Landsat TM and ETM+ imagery. *Remote Sensing of Environment*, 146, 136-147
50. Zhang, M., Tang, J., Dong, Q., Song, Q., & Ding, J. (2010). Retrieval of total suspended matter concentration in the Yellow and East China Seas from MODIS imagery. *Remote Sensing of Environment, 114*, 392-403
